# The Effects of a Cognitively Challenging Physical Activity Intervention on School Children’s Executive Functions and Motivational Regulations

**DOI:** 10.3390/ijerph191912742

**Published:** 2022-10-05

**Authors:** Athanasios Kolovelonis, Caterina Pesce, Marios Goudas

**Affiliations:** 1 Department of Physical Education and Sport Science, University of Thessaly, 42100 Trikala, Greece; 2 Department of Movement, Human and Health Sciences, Italian University Sport and Movement “ForoItalico”, 00135 Rome, Italy

**Keywords:** physical education, inhibition, cognitive flexibility, long-term intervention, retention

## Abstract

This study examined the effects of a physical education intervention consisting of cognitively challenging physical activity games on school children’s executive functions and motivational regulations. Ninety-nine fourth- and fifth-grade children participated in this two-group, repeated measures, quasi-experimental study with a cross-over design. Children’s executive functions (measured with the design fluency and Stroop and flanker tests) and motivational regulations were measured pre- and post-intervention and one month later. At post-test, the experimental group children outperformed the waiting-list control group children in all design fluency test conditions and accuracy in the Stroop and flanker tests. Both groups improved from pre- to post-intervention their speed (reaction time) in the Stroop and flanker tests. The waiting-list control group children, after receiving the intervention, improved their performance in the executive function tests except for Stroop test accuracy and flanker test speed. The positive effects were reduced significantly one month after the end of the intervention but remained significantly higher compared to pre-intervention. No intervention effects were found for the motivational regulations. These results showed that the intervention had positive effects on children’s executive functions and supported the new shift of designing physical activity programs for developing combinedly children’s physical and cognitive development.

## 1. Introduction

One important aim of school physical education is to promote physical activity, influencing children’s decisions to adopt physical activity for a lifetime [1]. At the same time, there is a need for implementing programs reflecting a balanced educational emphasis on learning skills and sports through innovative physical activities [2]. In this line, designing and implementing physical education interventions for pursuing multiple goals, including the enhancement of both children’s physical and cognitive development, is of great interest. 

Physical activity programs both in- and out-of-school should adopt a holistic approach to children’s development as an investment in human physical, cognitive, and socio-emotional capital [3,4], thus focusing not only on health-related outcomes and energy expenditure but also on cognitive development [5]. Position stands of authoritative institutions highlighted the value of physical activity to “fill two needs (i.e., cognitive and physical development) with one deed” [6]. Seminal works on the cognitive development benefits elicited by physical activity initially followed the search for dose–response relations that apply to the effects of physical activity on physical health and fitness [6,7]. Later on, it was proposed a shift from focusing only on the “quantity” of physical activity to increasing the “quality” of physical activity, emphasizing that features of the physical activity tasks as the coordinative and cognitive task complexity may generate cognitive engagement and thus, be independent of or interact with the physical and metabolic demands, responsible for the observed cognitive benefits [8,9,10]. This approach calls for increasing children’s physical activity shifting “from simply moving to moving with thought” [11] (p. 1).

The ongoing discussion on which type of physical activity is useful to reap the largest cognitive benefits has especially focused on executive function [12,13,14]. Executive functions refer to a family of higher-order cognitive processes responsible for cognitive flexibility and adaptability of goal-oriented behavior triggered in novel, challenging, and complex situations. There is a consensus that there are three core executive functions, namely inhibition, working memory, and cognitive flexibility [15]. Inhibition enables children to control attention, behavior, thoughts, and emotions for taking the most appropriate action, overriding a strong internal predisposition or blocking habitual or unsuitable actions. For example, a student inhibits an automated response because the conditions of the task require them to act in a totally different way. Working memory refers to the short-term storage and handling of information needed for effective actions. It helps children to interrelate and reorganize pieces of information, to translate instructions, or incorporate new information into action plans. Cognitive flexibility allows children to shift attention between task demands and change approaches to solve a problem by adjusting to new demands, rules, or priorities. It also helps them to think outside the box, approaching situations from a different point of view and taking advantage of sudden, unexpected opportunities.

Executive functions are essential for health, quality of life, wealth, and success in school and life [12]. Indeed, executive functions are considered important for school readiness [16], school success [17], and academic achievement [18]. Moreover, they are associated with success in sport [19] and mediate the relation between motor ability and academic achievement [20]. Executive functions are also associated with self-regulation and efficient metacognitive control [21] which in turn may lead to positive outcomes including improved motor and sport performance, satisfaction, and enjoyment [22].

Claims on the effects of physical activity quality and quantity on cognitive and particularly executive function development in children and youths are challenged by inconsistent conclusions of positive [6,23,24] vs. absence of effects [25,26]. This is likely due to a large array of moderators that act on the executive function–cognition relation, among which the quality and quantity of physical activity seem to play a relevant role across the lifespan [27] and specifically during development [28].

First attempts to quantify, meta-analytically, the effect sizes of different types of physical activity were performed, distinguishing between physical activity programs that were quantitatively enhanced (e.g., higher intensity, duration, or frequency) or qualitatively enriched (e.g., with cognitive task demands) [23]; between programs focused on aerobic training vs. cognitively challenging physical activity [29], also adding a further category of programs focused on motor skill acquisition, or combinations of aerobic, cognitively challenging, and motor skill interventions [30]. These first three reviews showed larger effect sizes for physical activities that were enriched with cognitive challenges. Two of them [23,30], which also performed separate analyses for different outcome measures, also revealed that among the three core executive functions, inhibition mostly benefited from physical activity programs characterized by cognitive enrichment and motor skill acquisition demands. Ludyga et al. [31] also reviewed the literature to further our understanding of the nuances of the executive function–cognition relation during development and found that the quantitative (intensity) and qualitative (coordinative complexity) characteristics of the physical activity programs interactively influenced working memory development.

The issue of physical activity enrichment has been reviewed also specifically in regard to interventional research performed in the physical education context [32]. Even though this review did not focus specifically on executive function, it corroborates the conclusion that quality-based physical education interventions increase cognition, mainly in primary education settings. However, the operationalization of “quality-based” physical education included both enriched physical education lessons with teaching strategies that were cognitively challenging and lessons expanded with high-intensity activities. Although this does not allow to disentangle quality and quantity, the quality-based physical education study with the largest effect size that most largely influenced the overall effect was one grounded in a method where increasingly more complex motor, cognitive, and motor–cognitive problems were provided for the learner to solve [33].

In sum, although physical activity programs that simply increase the amount of energy expenditure without any other cognitive demand seem to have limited effects on executive functions [13], it is still an issue of debate which combination of qualitative features of physical activity may be most beneficial. Diamond and Ling [13] have suggested that neither the metabolic nor the cognitive demands of physical activity per se may be best suited to reap the largest cognitive benefits unless they are associated with other features, such as mindfulness, positive affect, motivational value, and social interaction.

Thus, an ecological way to capitalize not only on the benefits of combining motor and cognitive demands in the same task [34] but also on the contribution of enjoyment and social interaction to foster executive function development [27], is to design cognitively challenging physical activity games. These games can be designed to adopt the approach of “moving with thought” [11] to increase children’s energy expenditure while involving them in cognitively challenging conditions [5]. They also engage children in unpredictable, changing, and complex conditions, requiring problem-solving and divergent discovery and offering space for adaptations and exploration about how to play the game or what strategies to apply [35]. They set challenges and mental demands involving children in novel and not highly repetitive or automatized tasks. The development of these games follows the principles of highlighting contextual interference, emphasizing mental control, and promoting discovery [35]. Contextual interference is created when the context and the game conditions change requiring children to make unpredictable sequences of actions. Mental control can be developed by stopping games (children must react in alternating signals to go and stop overriding prior actions), updating games (memory demands for holding and manipulating information are required), and switching games (children must stop their actions and act in a totally different way). For promoting discovery, children should be involved in conditions requiring divergent discovery (multiple solutions to a problem) and in open-ended games (the rules and the goal are explained, but not how to perform the game) [35].

Recently, preliminary evidence regarding the acute effects of these games has been reported. In particular, Kolovelonis and Goudas [36] found that three different types of games based respectively on the principles of highlighting contextual interference, emphasizing mental control, and promoting discovery had positive effects on children’s executive functions. Moreover, Kolovelonis and Goudas [37] found that children who were involved in such games improved their scores in executive functions and reported higher scores on novelty compared with children who were taught soccer or track and field skills. Similarly, these games were more effective in improving children’s executive functions and enhancing their total interest and instant enjoyment compared with traditional activities for enhancing their health-related fitness components [38]. This evidence suggested that those games are an appropriate means for enhancing children’s executive functions transiently. Since, however, partially different mechanisms underlie acute, transient, and chronic longer-term physical activity effects on cognitive development [28], it is relevant to apply these games with a chronic intervention design to test their effect on executive function and its maintenance.

### The Present Study

Cognitive, physical, emotional, or social development should be addressed jointly through appropriately designed physical education programs [39]. Thus, this study examined the effectiveness of an intervention with cognitively challenging physical activity games on triggering school children’s executive functions. Preliminary evidence regarding the acute effects of cognitively challenging physical activity games on school children’s executive functions has been reported [36,37,38]. However, further evidence from longer applications regarding the effects of these games is warranted. Previous respective studies combined physical activity games with other types of content such as playful forms of the variability of practice in physical activity [40], team sports (i.e., floorball and basketball) with a high level of cognitive engagement [41], and a cognitively enriched sports program [42]. Similar to the present study, Schmidt et al.’s intervention [41] also referred to the teaching principles developed by Tomporowki et al. [35] to challenge executive function. Although these interventions had positive effects on children’s executive functions, they embedded cognitive challenges in deliberate play [40] or sports games [41,42] that inherently elicit cognitive engagement. Thus, no conclusions can be inferred about the potential unique effects of cognitively challenging physical activity games on children’s executive functions. Thus, following suggestions for designing and implementing interventions focusing exclusively on specific activities to elucidate whether and how those programs benefit children’s executive functions [30], this study examined the effects of an intervention that consisted exclusively of cognitively challenging physical activity games.

Expanding previous research and strengthening the design of this study, a waiting-list control group and a retention measure were included. The implementation of the intervention with the waiting-list control group allowed the replication of the results providing stronger research evidence, whereas those children would also benefit from their involvement in this intervention. Moreover, it allowed counterbalancing the eventual influence of belonging to different schools on the intervention effects on executive function. Regardless of the positive effects of some types of physical activity on children’s executive functions, very little is known about the retention of these effects [43]. For example, Dalziell et al. [33] found significant effects of a novel physical education intervention (targeting the development of physical competence, cognitive skills, and personal qualities) on cognition (including inhibitory control and working memory) and coordination with the benefits maintained six months later. Thus, the present study was also targeted to examine retention effects, as recommended [13,43].

Motivating children to participate in physical education and to experience enjoyment and fun can prepare them for a lifetime of physical activity [44]. However, very little is known regarding the effects of cognitively challenging physical activity games on motivational variables. Some preliminary evidence has suggested that these games can have positive effects on children’s situational interests [37,38]. Expanding this research, the present study examined the effects of the intervention on children’s motivational regulations for participating in physical education. Enhancing children’s motivation through appropriately designed physical education programs can have positive outcomes including increased levels of motivation for physical activity outside of school [45].

This study examined the effects of an intervention consisting of cognitively challenging physical activity games on school children’s executive functions and motivational regulations. It was hypothesized that the children of the initial experimental group who would receive the intervention would outperform the wait-list control group children on executive functions. Similarly, the wait-list control group children would improve their scores in executive functions after receiving the intervention in the second phase of the study. It was also expected that children would retain, to some extent, their improvements in executive functions over time. Positive effects of the intervention on children’s motivational regulations were also expected.

## 2. Materials and Methods

### 2.1. Design

A two-group, repeated measures, cross-over quasi-experimental design was used in this study. The intervention was first implemented with the initial experimental group and after the post-test, the waiting-list control group received the intervention. The design involved pre- and post-intervention measures as well as retention measures one month after the intervention for executive functions and motivational regulations (Table 1).

### 2.2. Participants and Settings

Participants were 99 children (*M* age = 9.37 years old, *SD* = 0.59, 50 boys, 49 girls) from three fourth-grade (51 children, 24 boys) and three fifth-grade (48 children, 26 boys) classes of two elementary schools located in a medium-sized city in central Greece. The classes of one school (i.e., two fourth- and two fifth-grade) were assigned to the initial experimental group (61 children, 27 boys), and the classes of the second school (i.e., one fourth- and one fifth-grade) were assigned to the waiting-list control group (38 children, 23 boys). This study was conducted in real-life physical education settings and thus we involved intact physical education classes. For this reason, no exclusion criteria were used except for the requirement that children have written parental consent to participate in the study. All children provided parental consent to participate and thus all students involved were in the experiment. The intervention was implemented in the schools’ outdoor sport facilities.

The Greek physical education curriculum for grades 4 and 5 includes team sports (i.e., basketball, volleyball, soccer, and handball), individual sports (i.e., track and field and gymnastics), and traditional dance, and it is delivered by specialized physical education teachers in three 45-min sessions per week for fourth-grade children and two 45-min sessions per week for fifth-grade children.

### 2.3. Measures

#### 2.3.1. The Design Fluency Test

The design fluency test is part of the Delis–Kaplan Executive Function System which is a set of standardized tests for assessing executive functions showing satisfactory convergent and discriminant validity [46]. This test assessed children’s ability to draw as many different designs as possible in a predefined time (i.e., 60 s) generating novel designs by connecting dots with a pencil using four consecutive straight lines as quickly as possible while avoiding repeated patterns. The test included three conditions and a sheet with 35 square boxes with unstructured arrays of dots was used in each condition. In condition 1, each box contained five solid dots and children had to generate as many novel designs as possible using four consecutive straight lines. This condition measures design fluency. In condition 2, each box contained five solid and five blank dots and children had to generate as many novel designs as possible using four consecutive straight lines connecting only blank dots. This condition measures response inhibition as children had to use only blank dots avoiding the solid ones. In condition 3, each box contained five solid and five blank dots and children had to generate as many novel designs as possible using four consecutive straight lines alternating between connecting a solid and a blank dot (in each design they could start either from a solid or a blank dot). This condition measures the generation of novel designs while switching (cognitive flexibility). The number of correct and unique designs was the children’s score in each condition of the test. Before each test condition, children received respective instructions, they observed the experimenter performing one trial on the classroom blackboard, and then they performed a trial including three boxes of dots. A total score combining the scores in the three conditions was also calculated. Moderate test–retest reliability has been reported for the design fluency test [46].

#### 2.3.2. The Stroop Test

A computerized Stroop test for measuring inhibition was developed and administrated with E-Prime^®^ 3.0 software (Psychology Software Tools, Sharpsburg, PA, USA) (http://www.psnet.com (accessed on 21 July 2022)). The names of three colors (blue, green, and red), written in capital letters, Consolas font, size 36, were presented individually and randomly in the center of the screen on a white background. The test consisted of 50 words (16 writing red, 17 writing blue, 17 writing green) printed in colors that did not correspond to the written word (e.g., the word “green” written in blue letters) with the exception of one word of each color that corresponded to the written word (e.g., the word “green” written in green letters). Before the presentation of the words on the screen, a fixation (+) appeared for 0.5 s. Stimulus duration, that is the maximum time each word remained visible on the screen, was three seconds. If a student did not provide an answer within this time the next word appeared on the screen. Children had to select, as quickly as possible, the correct answer, which was the word presented on the screen regardless of the color printed, pressing, on a standard keyboard, button one for red, button two for green, and button three for blue. Before the test, children received instructions presented on the screen of the laptop and were analyzed by the experimenter. Then, they performed a trial consisting of nine words. In this trial, after each response children received feedback presented on the screen regarding the correctness of their answer, their reaction time, and the current percentage of correct answers. During the test, children did not receive any feedback. Children’s scores in the Stroop test were the number of correct answers (i.e., accuracy) and the mean score of their reaction time (i.e., speed) for the correct answers.

#### 2.3.3. The Flanker Test

A computerized modified version of the Eriksen flanker task [47] for measuring inhibition was developed and administrated with E-Prime^®^ 3.0 software (Psychology Software Tools, Sharpsburg, PA, USA) (http://www.psnet.com (accessed on 21 July 2022)). The test included the presentation on the center of the screen of a target array consisted of five arrows. Children had to respond quickly and accurately in the direction of the central arrow in congruent and incongruent conditions. In the congruent condition, the central arrow faced the same direction with the surrounding arrows (e.g., ← ← ← ← ← or → → → → →) whereas in the incongruent condition the central arrow faced the opposite direction with the surrounding arrows (e.g., ← ← → ← ← or → → ← → →). The flanker test was performed on compatible and incompatible stimulus–response conditions. In the compatible condition children were instructed to press the key that represented the direction of the central arrow. That is, when the central arrow faced right, children had to press the arrow (→) and when the central arrow faced left, children had to press the arrow (←). In the incompatible condition children had to press the key that represented the opposite direction of the central arrow. That is, when the central arrow faced right, children had to press the arrow (←) and when the central arrow faced left, children had to press the arrow (→). Before the presentation of the target array in the screen, a fixation (+) appeared for 0.5 s. The stimulus duration, that is the maximum time each target array remained visible on the screen, was 2 s. If children did not provide an answer within this time the next target array appeared on the screen. Two consecutive blocks (one for compatible and one for incompatible condition) of 48 attempts each were performed. In each block, half of the attempts were in congruent condition and half in incongruent. Before each test condition, children received instructions presented on the screen of the laptop and analyzed by the experimenter and performed a trial consisting of eight target arrays. In this trial, after each response, children received written feedback presented on the screen regarding the correctness of their answer, their reaction time, and the current percentage of correct answers. During the test, children did not receive any feedback. Children’s scores in the flanker test were the total number of correct answers (i.e., accuracy) and the mean score of their reaction time (i.e., speed) for the correct answers.

#### 2.3.4. Motivational Regulations

The Greek version [48] of the Motivational Orientations Questionnaire [49] was used. The questionnaire evaluates children’s reasons for participating in physical education. It begins with the stem “I participate in physical education lesson….” and consists of 5 subscales: intrinsic motivation (4 items; e.g., “…because it is enjoyable”), identified regulation (4 items; e.g., “…because it is important for me to do well in the lesson”), introjected regulation (4 items; e.g., “…because it would bother me if my teacher though that I am not a good student”), external regulation (3 items; e.g., “…because this is mandatory”), and amotivation (4 items; e.g., “… I really don’t know why”). Responses were rated on a 7-point Likert scale (1 = strongly disagree, 7 = strongly agree). Internal consistency of the five questionnaire subscales was satisfactory (Cronbach’s α ranged from 0.65 to 0.83). Children’s scores in the five questionnaire subscales were used as dependent variables. Moreover, the relative autonomy index was calculated for each student based on the relative weight of the five motivational regulations (intrinsic, identified, introjected, external, and amotivation) according to their place in the self-determination continuum (+2, +1, −1, −2, −3, correspondingly).

### 2.4. Procedures

Ethical approval for the study was granted by the Ministry of Education and the University Ethics Review Committee (blinded for review). Permissions were also obtained from the school principals and the physical education teachers. Children participated voluntarily after written parental consent was obtained. The intervention was delivered by an experimenter, blind to the aims of the study, who was a physical education teacher with a Master’s degree in physical education and experience in implementing physical education interventions. All sessions were conducted in the schools’ open sport facilities during regular physical education hours. Written detailed lesson plans were developed for the intervention sessions and were piloted with classes of children not participating in this study. After each session, the experimenter kept notes about the fidelity of the implementation and children’s involvement in the session. All plans were implemented as planned and children of all groups were actively involved in each session. One week before the intervention, children were pre-tested completing the design fluency test and the motivational regulations questionnaire in their classroom. Next, children in pairs participated in the computerized Stroop and flanker tests in a quiet classroom with the presence of the experimenter. Two laptops with a 15.6-inch color high-definition monitor were used and placed at the level of children’s eyes at a distance of approximately 50 cm and two meters apart in a way precluding children to see each other’s screen. Before each test, children received appropriate instructions and performed a trial (see details in the measure section). After the intervention, all children were post-tested following the procedures used in the pre-test. Next, the intervention was implemented with the waiting-list control group children and after that, children were tested again following similar procedures. This third measure was the retention measure for the initial experimental group children and the post-intervention measure for the waiting-list control group children. One month later, a retention measure was also implemented with the waiting-list control group children.

### 2.5. Description of the Intervention

The intervention consisted of eight 45-min physical education sessions delivered over a period of four weeks (i.e., two sessions per week). Each session included 4–6 games. A 3-min organizational period at the beginning of each session and a 3-min closure and reflection section at the end of each session were also included. The physical activity games included in the intervention highlighted contextual interference, mental control, and discovery [35]. In particular, the conditions of some games were changing continuously requiring children to respond to unpredictable sequences of actions, or in roles with various demands. The rules were altered appropriately to make games more challenging. For example, in some games, children should move following the instruction of the teacher whereas in the next phase of the game they should do the opposite. Some games set challenges to children’s working memory by requiring them to hold information and manipulate them appropriately during the game. Other games required children to inhibit their regular actions and act in a totally different way or to override prior actions. Some other games involved children in open-ended problem conditions requiring them to produce multiple and unique solutions or select the most appropriate actions or strategies. Examples of games included in the intervention were hop, pop, and tag, modified crazy traffic lights, maps, tag in groups, soccer with two balls, soccer with two balls and four goals, do what I do or the opposite, and photo-copy [35,50]. A detailed description of these games is presented in Appendix A. Moreover, a description of the implementation of the intervention, including the teaching strategies used and how the criteria of contextual interference, mental control, and discovery were implemented in specific games, is presented in Supplementary File S1. A detailed and in-depth analysis of two sample games [40] is presented in Supplementary File S2. For a similar approach, with younger children, see Biino et al. [51]. In the first phase of the study, the waiting-list control group of children followed the school’s physical education program. In the second phase, they received the intervention including the same games used in the initial experimental group of children.

### 2.6. Statistical Analysis

The effects of the intervention on the initial experimental group children’s scores in the three conditions of the design fluency test and in the Stroop test scores (i.e., accuracy and reaction time) were examined with separate 2 (Groups, initial experimental, and initial control) × 2 (Times, T_1_ and T_2_) repeated measures MANOVAs followed by univariate analysis (repeated measures ANOVAs) and pre-test to post-test comparisons within each group. For the flanker test (i.e., accuracy and reaction time), due to pre-test differences between groups (see Section 3.1), the effects of the intervention on the initial experimental group children’s scores were examined through a MANCOVA with Group as factor, T_2_ accuracy, and reaction time as the dependent variable and T_1_ pre-test scores serving as covariates. Intervention effects on the waiting-list control group children’s scores in the executive functions (i.e., design fluency test, Stroop test, flanker test) were examined with pre- and post-intervention comparisons through repeated measures MANOVAs. To examine the compound effects of the intervention implemented in both groups (i.e., initial experimental and initial control group) and the retention of these effects, a pre-intervention (T_1_ for the experimental group and T_2_ for the control group), a post-intervention (T_2_ for the experimental group and T_3_ for the control group), and a retention score (T_3_ for the experimental group and T_4_ for the control group) of children’s executive function served as dependent variables in repeated measures MANOVAs. A similar pattern of analyses was used for examining the intervention effects on children’s motivational regulations. Effects sizes of partial *η^2^* and Cohen’s *d* were also calculated [52].

## 3. Results

### 3.1. Preliminary Analyses

A post hoc power analysis (G*power) was conducted to compute the achieved power for the Group × Time interaction of interest, with sample size = 99, a 2 (Group) × 2 (Time) Repeated Measures ANOVA, *p* < 0.05, effect sizes of the Group × Time interaction *f* [√ η^2^p/(1 − η^2^p)] and correlations (*r*) between repeated measures for each individual outcome variable. The achieved power (1 − β) was very high for all conditions and the total score of the design fluency test and the correct answers in the Stroop test (0.99, 1.00, 1.00, 1.00, and 0.99, respectively) and very low for the reaction time in the Stroop test (0.05). The achieved power for the one-way ANCOVA used in the flanker test was 0.50 and 0.56, for the correct answers and the reaction time, respectively.

Data were generally normally distributed with some exceptions in the third condition of the design fluency test and the accuracy in the Stroop and the flanker test in Time 2 and 3. Means and standard deviations for children’s scores in the executive function tests are presented in Table 2. No pre-test differences between groups were found in the three conditions of the design fluency test, *F*(3, 94) = 0.43, *p* = 0.731, in the total score of the design fluency test, *t*(96) = 0.63, *p* = 0.529, in the accuracy, and in the reaction time in the Stroop test, *F*(2, 96) = 2.17, *p* = 0.120. Significant pre-test differences were found between groups in the flanker test, *F*(2, 96) = 5.77, *p* = 0.004, *η*^2^ = 0.107. The waiting-list control group children scored significantly higher compared to the initial experimental group children in the accuracy, *F*(1, 97) = 10.07, *p* = 0.002, *η*^2^ = 0.094, and in the reaction time *F*(1, 97) = 5.20, *p* = 0.025, *η*^2^ = 0.051. No pre-test differences between groups were found on the five motivational regulations, *F*(5, 92) = 1.84, *p* = 0.112. However, the waiting-list control group children reported higher pre-test scores in the relative autonomy index compared with the initial experimental group children, *t*(96) = 2.87, *p* = 0.005.

### 3.2. Design Fluency Test

A significant multivariate Group × Time interaction, *F*(3, 93) = 10.74, *p* < 0.001, *η*^2^ = 0.257, on children’s scores in the three conditions of the design fluency test was found. Univariate tests showed a significant Group × Time interaction for test condition 1 (fluency), *F*(1, 95) = 7.11, *p* = 0.009, *η*^2^ = 0.070, test condition 2 (inhibition), *F*(1, 95) = 15.26, *p* < 0.001, *η*^2^ = 0.138, and test condition 3 (flexibility), *F*(1, 95) = 26.97, *p* < 0.001, *η*^2^ = 0.221. Pre- to post-test improvements were found for the experimental group in all test conditions, but for the waiting-list control group in condition 1 only (Table 3). The 2 (Group) × 2 (Time) ANOVA showed a significant Group × Time interaction, *F*(1, 95) = 27.70, *p* < 0.001, *η*^2^ = 0.223, on children’s total score in the design fluency test. Follow-up comparisons showed significant improvements with different effect sizes from pre-test to post-test for the experimental group and for the waiting-list control group (Table 3).

Regarding the waiting-list control group children’s T_2_ and T_3_ scores on the design fluency test, the MANOVA showed a significant effect for Time, *F*(3, 35) = 21.24, *p* < 0.001, *η*^2^ = 0.645. Univariate tests showed that the waiting-list control group children improved their scores from T_2_ to T_3_ in all test conditions and in the total design fluency score, *t*(37) = 7.65, *p* < 0.001, *d* = 1.02 (Table 3).

As regards the compound effects of the intervention implemented in both groups and the retention of these effects, a significant multivariate effect for Time was found, *F*(6, 368) = 28.99, *p* < 0.001, *η*^2^ = 0.321. Univariate tests showed a significant effect for test condition 1, *F*(2, 182) = 41.72, *p* < 0.001, *η*^2^ = 0.310, test condition 2, *F*(2, 182) = 53.71, *p* < 0.001, *η*^2^ = 0.366, and test condition 3, *F*(2, 182) = 52.14, *p* < 0.001, *η*^2^ = 0.359. Pairwise comparisons showed that in all three test conditions, children improved significantly their scores from pre-test to post-test (condition 1: *p* < 0.001, *d* = 0.93; condition 2: *p* < 0.001, *d* = 0.98; condition 3: *p* < 0.001, *d* = 1.22), and despite the significant reduction from post-test to retention (condition 1: *p* = 0.012, *d* = 0.29; condition 2: *p* < 0.001, *d* = 0.51; condition 3: *p* < 0.001, *d* = 0.74), the improvement from pre-test to retention remained significant (condition 1: *p* < 0.001, *d* = 0.61; condition 2: *p* < 0.001, *d* = 0.46; condition 3: *p* = 0.026, *d* = 0.32). Similarly, the one-way ANOVA performed on the total design fluency score showed a significant effect for Time, *F*(2, 92) = 86.47, *p* < 0.001, *η*^2^ = 0.653. Pairwise comparisons showed the same pattern of pre-to-post improvement (*p* < 0.001, *d* = 1.24) that despite the significant reduction from post-test to retention (*p* < 0.001, *d* = 0.62), remained significant from pre-test to retention (*p* = 0.026, *d* = 0.57).

### 3.3. Stroop Test

A significant multivariate Group × Time interaction was found, *F*(2, 96) = 5.28, *p* = 0.007, *η*^2^ =.098. The univariate tests showed a significant Group × Time interaction for accuracy in the Stroop test, *F*(1, 97) = 9.92, *p* = 0.002, *η*^2^ = 0.093. Significant improvements from pre-test to post-test were found for the experimental group but not for the waiting-list control group (Table 3). Group × Time interaction for the reaction time in the Stroop was nonsignificant, *F*(1, 97) = 0.006, *p* = 0.939, but a significant effect for Time was found, *F*(1, 97) = 19.37, *p* < 0.001, *η*^2^ = 0.166. Both the initial experimental group and the waiting-list control group children improved their reaction time from pre-test to post-test (Table 3).

Regarding the waiting-list control group children’s pre- (T_2_) and post-intervention (T_3_) performance (i.e., accuracy and reaction time), the MANOVA showed a significant Time effect, *F*(2, 35) = 10.47, *p* < 0.001, *η*^2^ = 0.374. Univariate tests showed a pre-to-post improvement in reaction time, but the improvement in accuracy did not reach significance (Table 3). As regards the compound effects of the intervention implemented in both groups and the retention of these effects, a significant effect for Time was found in the MANOVA performed on accuracy and reaction time data, *F*(4, 362) = 20.36, *p* < 0.001, *η*^2^ = 0.184. Univariate tests showed a significant effect for accuracy, *F*(2, 182) = 23.91, *p* < 0.001, *η*^2^ = 0.208, and for reaction time, *F*(2, 182) = 20.31, *p* < 0.001, *η*^2^ = 0.182. For accuracy, pairwise comparisons showed that children improved significantly from pre-test to post-test (*p* < 0.001, *d* = 0.85), and despite the significant reduction from post-test to retention (*p* = 0.021, *d* = 0.35), the improvement from pre-test to retention remained significant (*p* < 0.001, *d* = 0.48). For reaction time, pairwise comparisons showed that children improved significantly from pre-test to post-test (*p* < 0.001, *d* = 0.43) and further improved from post-test to retention (*p* = 0.020, *d* = 0.26).

### 3.4. Flanker Test

The effects of the intervention on the initial experimental group children’s scores in the flanker test (i.e., accuracy and reaction time) were examined with a MANCOVA, with pre-test scores serving as covariates to adjust for group differences at pre-test. There was a significant difference between groups, *F*(2, 94) = 4.51, *p* = 0.014, *η*^2^ = 0.087. The initial experimental group of children outperformed in the post-test the waiting-list control group in the accuracy in the flanker test, *F*(1, 95) = 3.76, *p* = 0.056, *η*^2^ = 0.038, whereas the waiting-list control group children had better reaction time compared with the experimental group in the post-test, *F*(1, 95) = 4.36, *p* = 0.040, *η*^2^ = 0.044.

Regarding the waiting-list control group children’s pre- (T_2_) and post-intervention (T_3_) performance (i.e., accuracy and reaction time), a repeated measures MANOVA showed a significant effect, *F*(2, 35) = 9.35, *p* < 0.001, *η*^2^ = 0.348. Univariate tests showed that waiting-list control group children improved significantly, from pre- to post-intervention, their accuracy, *F*(1, 36) = 8.07, *p* = 0.007, *η*^2^ = 0.183, but not their reaction time, *F*(1, 36) = 2.59, *p* = 0.117.

As regards the compound effects of the intervention implemented in both groups and the retention of these effects, significant multivariate effect for Time was found, *F*(4, 362) = 27.57, *p* < 0.001, *η*^2^ = 0.234. Univariate tests showed a significant effect for accuracy, *F*(2, 182) = 31.93, *p* < 0.001, *η*^2^ = 0.256, and for reaction time, *F*(2, 182) = 31.14, *p* < 0.001, *η*^2^ = 0.255. For accuracy, pairwise comparisons showed that children improved significantly from pre-test to post-test (*p* < 0.001, *d* = 0.95), and despite the significant reduction from post-test to retention (*p* = 0.043, *d* = 0.29), the improvement from pre-test to retention remained significant (*p* < 0.001, *d* = 0.61). Moreover, children improved significantly their reaction time from pre-test to post-test (*p* < 0.001, *d* = 0.33) and further improved from post-test to retention (*p* < 0.001, *d* = 0.31).

### 3.5. Motivational Regulations

Neither the 2 (Groups) × 2 (Times) MANOVA applied to T_1_ and T_2_ data of both groups on motivational regulations, *F*(5, 90) = 1.07, *p* = 0.384, nor the ANCOVA with the adjustment for group differences at pre-test in the relative autonomy index *F*(1, 93) = 0.131, *p* = 0.719, yield significant results. In addition, the MANOVA applied to T_2_ and T_3_ data of the waiting-list control group did not yield any significant results on students’ motivational regulations, *F*(5, 32) = 0.20, *p* = 0.961. Similarly, nonsignificant difference were found on relative autonomy index, *t*(36) = 0.43, *p* = 0.669.

## 4. Discussion

This study examined the effects of an intervention with cognitively challenging physical activity games on school children’s executive functions and motivational regulations. Generally, the results showed that, after receiving the intervention, children improved in executive function performances including their scores in the design fluency test and in accuracy in the Stroop and the flanker tests. Most importantly, these effects were partially retained one month later compared to the pre-intervention performance. No intervention effects were found on children’s motivational regulations. These results are discussed with reference to previous evidence and with an outlook for designing physical activity programs focusing on children’s physical and cognitive development jointly.

The results showed significant improvements after the intervention in children’s executive functions. Indeed, the generally positive impact of the intervention on executive function is proven by the fact that the initial experimental group of children improved significantly more than the waiting-list control group in each of the three conditions and in the total score of the design fluency test. The cross-over design strengthens this finding, allowing us to exclude that the beneficial effects are due to the differential responsiveness of experimental and control groups to an intervention. Moreover, although the existence of a learning effect between pre- and post-test cannot be excluded, the difference in the effects between the experimental and the wait-list control group was generally high, denoting strong effects on children’s executive function. These results expanded previous evidence regarding the transient beneficial effects of an acute bout of these physical activity games on children’s executive function assessed with the same test [36,37,38], by showing that a longer intervention with these games can positively affect children’s executive functions in the longer term.

The effects of the intervention on the Stroop and flanker tests were generally positive but some mixed results were also found, suggesting that the mixed evidence that led to meta-analytically modest effect sizes [53] may be due not only to differences in quantitative and qualitative characteristics of the interventions [8] but also to the different type of individual tests used to assess a specific function in different studies [54]. After the intervention, the initial experimental group of children outperformed the waiting-list control group of children in performance accuracy in the Stroop and the flanker tests, but not in reaction speed, which improved in both groups. Moreover, after receiving the intervention, the waiting-list control group of children improved their scores in all executive function variables with the exception of their accuracy in the Stroop test and the reaction time in the flanker test. The lack of improvements in these performance measures may be explained by the fact that the waiting-list control group of children had already improved between the first and the second measure when they served as the control group participating in a regular physical education lesson. The significant enhancement of reaction speed in the tests tapping inhibition, coupled with a tendency of reduced accuracy, suggests that the effect of cognitively challenging games might reflect a shift in the speed-accuracy trade-off setpoint. If children exposed to enrichment in physical education can learn trading accuracy for speed, then further research is warranted.

Overall, these results are consistent with previous findings showing the positive effects on children’s executive functions of cognitively enriched physical activity interventions combining different types of physical activities, games, and sports [40,41,42,55]. The improvements of the comparison group (i.e., the waiting-list control group) which followed regular physical education sessions, in some of the executive function performances can be attributed to a possible learning effect. However, the effects were stronger for the experimental group, ranging from moderate to high, denoting that, apart from any possible learning effects, the cognitively challenging physical activity games had a unique impact on school children’s executive function. This also highlights the effects of the cognitively challenging physical activity game on triggering children’s executive functions against regular physical education and in contrast with other physical activity interventions without cognitive stimuli that have resulted in small or non-significant effects [30].

Recent reviews and meta-analyses [24,30] have suggested that although physical activity is a promising way to promote executive functions, not all physical activity interventions were effective, as the size of the effects was, in some cases, small or non-existent when compared to traditional physical education programs. What is critical is the nature of these interventions. The potential of physical activity interventions to have positive effects on children’s executive functions can be increased when the interventions are enriched with cognitive challenges [23,29,30,56]. The present study supported this view showing that a physical activity intervention consisting of cognitively challenging physical activity games had strong effects on children’s executive functions.

Cognitively enriched physical activity interventions may have different effects on the various aspects of executive functions. Indeed, previous studies have performed separate analyses for different outcome measures of executive functions showing that inhibition mostly benefited from cognitively enriched physical activity [23,30]. However, considering that not all evidence supported this trend [28], suggestions for expanding this line of research involving all aspects of executive functions have been recently reported [28].

In the present study, executive function was measured with three different tests (i.e., design fluency, Stroop and flanker tests). Instead of using, as commonly conducted in physical activity and cognition research, three different tests to tap the three core executive functions (inhibition, working memory, and cognitive flexibility), we chose a set of tests that converged on inhibition. Indeed, this is the most-studied executive function in developmental exercise and cognition research, with meta-analytical evidence of overall only small effect sizes for performance accuracy [51], but most pronounced effects when physical activity is cognitively challenging and skill demanding [23,30]. The use of multiple inhibition tests allowed us to confirm, with an in-depth exploration, that inhibition is sensitive to cognitively challenging physical activity. However, our physical activity games were tailored also to challenge other executive functions needed, for example, to solve motor problems. Thus, we included in our battery also a test involving a diverse set of measures that could be analyzed individually, as well as in a composite manner (total score) to tap shared variance across outcome measures [57]. This allowed us to generalize the benefits of our games to the broader executive function construct without renouncing an in-depth view of specific executive functions of interest. Moreover, regarding inhibition, accuracy (i.e., correct answers) and speed (i.e., reaction time) in the Stroop and the design fluency tests were analyzed separately. The results of this study supported previous evidence [23] that accuracy rather than speed is the performance component that is mostly improved by physical activity in children. However, these interpretations should be further explored in future research.

Most importantly, filling a gap in the field [13,43], this study examined the retention of the effects of the cognitively enriched physical activity intervention in physical education. The results showed that the positive effects of the intervention on children’s executive function were still partially maintained one month after the end of the intervention compared to the pre-intervention levels, despite a reduction between post-intervention and retention assessment times. These results add to previous evidence showing that benefits in children’s cognition (including inhibitory control) were maintained six months after the end of a cognitively enriched physical education intervention [33]. Thus, cognitively challenging physical activity games can enhance children’s executive functions and these effects remain significant at least one month later. A rather unexpected but intriguing result was that children’s reaction time in both the Stroop and the flanker tests were further improved after the post-intervention measure. Probably, children may have enjoyed these games a great deal and this may have led them to enjoy engaging in activities that are inherently cognitively challenging (such as team games) in their free time. Future research should explore if this result is replicable; if so, it might be that having reached a threshold level of improvement, children might be able to capitalize on it to further improve also in the absence of specifically tailored stimulations.

The cognitively challenging physical activity games seem to have the appropriate characteristics for enhancing children’s executive functions. Indeed, these games involve children in challenging, complex, and unpredictable conditions requiring mental effort and problem-solving [35]. For being successful in such play and learning environments, children are forced to put much more mental effort to memorize the requirements of the various roles or movement sequences, select appropriate actions, elaborate and think deeply about the features of the games, or exhibit creativity and cognitive flexibility [58]. Such cognitively challenging environments may activate brain regions used to control higher-order cognitive processes [39,59], thus facilitating the development of children’s executive functions [5].

No intervention effects on children’s motivational regulation were found. Children were already highly motivated for participating in physical education at the onset of the intervention reporting high scores in the adaptive motivational regulations (i.e., intrinsic motivation and identified regulation) and low scores on the external motivation and amotivation. Thus, room for improvement in children’s scores on these variables after the intervention was rather restricted. Indeed, previous findings have shown that elementary children are highly motivated to participate in physical education [60]. Nevertheless, enhancing children’s motivation should still be an important facet of all interventions and programs implemented in physical education. Enhanced children’s motivation is associated with positive outcomes including increased levels of motivation for physical activity outside of school [45]. The cognitively challenging physical activity games seem to have the appropriate characteristics for attracting children’s interest and providing them with fun. Indeed, previous evidence has shown that these games had positive effects on children’s situational interests [37,38]. However, these effects should be verified in future research involving long-term interventions.

This study was conducted in real-life physical education settings and therefore can inform important practical development. Physical educators can systematically include in their programs cognitively challenging physical activity games to enhance children’s executive functions. Traditional forms of physical activities including running or repetitive fitness routines may fail to attract some children’s interest and thus these children may be reluctant to actively be involved in such tasks [61]. Enriching their programs with cognitively challenging physical activity games, physical educators may be more successful in involving their children in physical activity programs.

Children’s physical activity during the intervention was not measured in this study. This limitation should be addressed in future research examining the effects of the different types of games on children’s physical activity. Children’s performance in these games may also be measured by examining the potential effects of this performance on their executive functions. Considering that this study involved a four-week intervention, future research should also examine the effects of larger interventions and the retention of the potential effects for longer periods of time. Such research may also involve boost sessions (e.g., two months after the end of the intervention) with cognitively challenging physical activity games to examine the effects of these additional sessions on the retention of the intervention effects. Future research may also compare the effects of these games with other physical education programs on children’s executive functions. Considering that a possible learning effect may exist in this study, future research may involve additional pre-test measures of children’s executive functions to control such effects. Moreover, it should be considered that this learning effect might be best represented by a deceleration curve. That is, after the first two assessments (before and after the one-month intervention), the children had already reached the plateau of their learning curve.

## 5. Conclusions

This study showed that an intervention consisting of cognitively challenging physical activity games improved school children’s executive functions. These effects were partially retained one month after the end of the intervention. These games seem to have the appropriate characteristics for enhancing children’s executive functions and can be used for designing and implementing cognitively enriched physical activity interventions in physical education settings.

## Figures and Tables

**Table 1:** The design of the study.

	Time 1	Intervention	Time 2	Intervention	Time 3	Time 4
	(Week 0)	(Week 1–4)	(Week 4)	(Week 5–8)	(Week 8)	(Week 12)
Experimental group	O	X	O		O	
Waiting-list control group	O		O	X	O	O

Note: Χ: intervention, O: measures.

**Table 2:** Means and standard deviations for children’s scores in all variables measuring executive functions in all time measures of the study, separate for the experimental and the waiting-list control group.

	Experimental Group						Waiting-List Control Group							
	Time 1		Time 2		Time 3		Time 1		Time 2		Time 3		Time 4	
Variable	*M*	*SD*	*M*	*SD*	*M*	*SD*	*M*	*SD*	*M*	*SD*	*M*	*SD*	*M*	*SD*
DF-condition 1	8.37	3.41	12.17	3.47	11.25	4.05	8.47	2.82	10.24	4.06	13.16	3.33	11.73	3.62
DF-condition 2	8.05	3.38	11.78	3.50	9.88	3.40	8.26	3.38	9.08	3.65	12.11	2.99	10.46	3.64
DF-condition 3	4.36	2.45	8.66	3.64	5.56	3.94	5.03	2.59	5.29	2.64	8.16	3.48	6.08	3.66
DF-total	20.78	7.48	32.61	8.82	26.68	9.30	21.76	6.81	24.61	8.95	33.42	8.24	28.27	8.66
Stroop-CA	30.26	14.24	44.11	7.60	38.75	12.44	36.74	16.06	40.95	14.05	45.11	8.15	43.85	8.41
Stroop-RT	1029.7	319.1	911.4	202.1	826.2	216.3	985.8	253.7	863.4	191.9	777.9	197.6	766.4	173.3
Flanker-CA	57.48	16.89	79.82	15.63	73.23	20.30	69.53	18.83	77.77	16.10	84.78	9.86	81.44	13.51
Flanker-RT	818.6	180.5	732.8	185.1	660.6	188.6	738.4	151.9	634.7	108.1	603.2	132.9	585.5	120.2

Note: DF: design fluency; Stroop-CA: correct answers in the Stroop test; Stroop-RT: reaction time in the Stroop test; Flanker-CA: correct answers in the flanker test; Flanker-RT: reaction time in the flanker test. Reaction times are given in milliseconds. T1: pre-intervention; T2: post-intervention (experimental group) and post-waiting (initial control group); T3: one-month follow-up after intervention cessation (experimental group) and post-intervention (control group after cross-over); T4: one-month follow-up after intervention cessation (control group).

**Table 3:** Univariate pre- to post-test comparisons separately for the experimental and the waiting-list control group, for the design fluency and Stroop test variables.

	Experimental Group			Waiting-List Control Group					
	T1–T2			T1–T2			T2–T3		
Variable	*t*	*p*	*d*	*t*	*p*	*d*	*F*	*p*	*η^2^*
DF-condition 1	8.34	0.001	1.11	2.78	0.009	0.51	22.86	0.001	0.382
DF-condition 2	9.08	0.001	1.08	1.21	0.236	-	36.69	0.001	0.498
DF-condition 3	7.96	0.001	1.39	0.54	0.590	-	30.74	0.001	454
DF-total	11.11	0.001	1.04	2.07	0.045	0.36	7.65 *	0.001	1.02 *
Stroop-CA	8.26	0.001	1.21	1.51	0.141	-	3.47	0.071	-
Stroop-RT	3.27	0.002	0.44	3.25	0.002	0.54	10.34	0.003	0.223

Note: * These values represent *t* and *d* scores, respectively. DF: design fluency; Stroop-CA: correct answers in the Stroop test; Stroop-RT: reaction time in the Stroop test. Reaction times are given in milliseconds. T1: pre-intervention; T2: post-intervention (experimental group) and post-waiting (waiting-list control group); T3: post-intervention for waiting-list control group after cross-over.

## Data Availability

The data underlying the results presented in the study are part of a research program and are available on request from the third author (M.G.; mgoudas@pe.uth.gr).

## Supplementary Materials

The following supporting information can be downloaded at: https://www.mdpi.com/article/10.3390/ijerph191912742/s1, File S1: Cognitively enriched physical activity intervention (Group 1), File S2: “Joy of Moving” sample games.

## Appendix A

### Examples of Cognitively Challenging Physical Activity Games Included in the Intervention

**Hop, pop, and tag**: Children try to tag any other student while avoiding being tagged. The tagged children squat down and can enter back into the game when their tagger is tagged by another student. This game challenges children to avoid being tagged by multiple taggers while they should tag other children. Moreover, tagged children should be aware if their taggers are tagged in order to return back into the game [35].

**Tag in groups**: Children of one team try to tag any other student of the other team while avoiding being tagged. The main rules of the hop, pop, and tag are followed.

**Modified crazy traffic lights**: Children perform specific movements or sequences of movements following instructions or signals provided by the teacher. Instructions and signals are given orally (e.g., three steps front and one step right), using numbers (e.g., 1: move forward, 2: move back, 3: move right, 4: move left), or colored cards (each color represents specific movement). The task gradually becomes more complex including a longer sequence of actions and a combination of signals. In a variation of this game, two contradictory signals are presented simultaneously (e.g., an oral signal for moving forward and a colored card for moving back) and children had to perform the action following the dominant signal set at the beginning of the game (e.g., colored card). This game challenges children to react appropriately to alternating signals and set memory demands for holding and manipulating information [50].

**Do what I do or the opposite**: This task is similar to modified crazy traffic lights. Children perform movements or sequences of movements following the various stimulus introduced by the teacher in the form of instructions (e.g., step right, step left, hop ahead, etc.). Next, a stimulus is presented in the form of numbers, colors, or in combination (e.g., number 1 represents a step right, number 2 a step left, etc.). Gradually, the movements become longer and more complex. Then, children are asked to perform the opposite action compared to those presented (e.g., if the teacher calls number 1 that means to step right, children should step left).

**Maps**: Children move back and forth in the playing area selecting their way and performing various movements or sequences of movements. At specified points within the playing area, there is equipment (e.g., hoops, ropes, balls) that children could use for performing their movements. In each trial, children had to follow a different route and perform different movements [35,50].

**Photo-copy**: Children in pairs. One student performs a movement or a shape and the other reproduces the action. Gradually, the movements become longer and more complex [50].

**How many different ways you can find to…?:** This stem is presented to the children followed by various tasks asking the children to produce multiple and novel solutions to problem-solving conditions. Examples of problem-solving tasks are to move with different movement patterns from one side to the other, to pass a ball to a teammate, to override an obstacle, etc.

**Soccer with two balls**: The regular rules of the soccer game are followed with the exception that two balls are introduced into the game. Each team may have in their possession one, both, or neither ball. This set challenges the children of each team to select the most effective strategies in both defense and offense.

**Soccer with two balls and four goals**: Similar to soccer with two balls but each team has to defend two goals.
