# Supplementary material for: The Effects of a Cognitively Challenging Physical Activity Intervention on School Children’s Executive Functions and Motivational Regulations
Source: Int J Environ Res Public Health. 2022 Oct 5;19(19):12742. doi: 10.3390/ijerph191912742 (PMC9564380; doi:10.3390/ijerph191912742)
Supplement: Supplementary file 1 [file ijerph-19-12742-s001.zip › Suppl_File_1.pdf]

Supplementary material 1.

Cognitively enriched physical activity intervention (Group 1)

[For a similar approach with younger children, see Biino et al., 2021]

**Content**	The content of the cognitively enriched physical activity intervention consisted of cognitively challenging physical activity games based on different principles derived from motor learning and cognitive stimulation theories and practices.
**Teaching strategies**	Central to the games was to generate and maintain mental effort at an optimal challenge point by progressively increasing the complexity of movement coordination and/or the demands on thoughtful action. To this aim, games can be varied by:<br>• Alternating the roles played children (e.g., roles of tagger and tagged children in tag games);<br>• Changing the motor demands by adding movements to a sequence, or increasing the coordinative difficulty of the single movements that compose a sequence;<br>• Altering the structure of a game to encourage problem solving and divergent discovery.
**Principles**<br><br>(described in detail in: Tomporowski, P. D., B. A. McCullick, and C. Pesce. 2015. Enhancing Children's Cognition with Physical Activity Games. Champaign, IL: Human Kinetics.	The **principle of contextual interference** set that the context and the game conditions change requiring children to make unpredictable sequences of actions and perform shifts across multiple skills, or variations of a skill. This way children may generate mental effort and challenge cognitive flexibility to select every time different motor programs and movement parameters.<br><br>The **principle of mental control** is operationalized in:<br>• stopping games or tasks, where children must on occasion refrain from or stop moving;<br>• updating games or tasks, where children must continuously update information held in memory to cope with ongoing movement tasks properly;<br>• switching games or tasks, in which perception-action couplings and criteria to perform movement tasks are frequently and suddenly changed).<br><br>The **principle of promoting discovery** is translated into practice by involving children in conditions requiring divergent discovery (generating multiple solutions to a problem) and in open-ended games (the rules and the goal are explained, but not how to perform the game).
**Sample games**	A description of sample games is presented in Appendix 1. A detailed and in-depth analysis of two sample games (Pesce et al., 2016) is presented in Supplementary File 2.<br>Pesce, C., Marchetti, R., Motta, A., & Bellucci, M. (eds.) (2016). Joy of moving - MindMovers & ImaginAction. Playing with variability to

	promote motor, cognitive and citizenship development. Torgiano (PG): Calzetti-Mariucci.

Biino, V., Tinagli, V., Borioni, F., & Pesce, C. (2021). Cognitively enriched physical activity may foster motor competence and executive function as early as preschool age: A pilot trial. Physical Education and Sport Pedagogy [online first]. https://doi.org/10.1080/17408989.2021.
