# Supplementary material for: The Effects of a Cognitively Challenging Physical Activity Intervention on School Children’s Executive Functions and Motivational Regulations
Source: Int J Environ Res Public Health. 2022 Oct 5;19(19):12742. doi: 10.3390/ijerph191912742 (PMC9564380; doi:10.3390/ijerph191912742)
Supplement: Supplementary file 1 [file ijerph-19-12742-s001.zip › Suppl_File_2.pdf]

Watch game videos online

Joy of moving

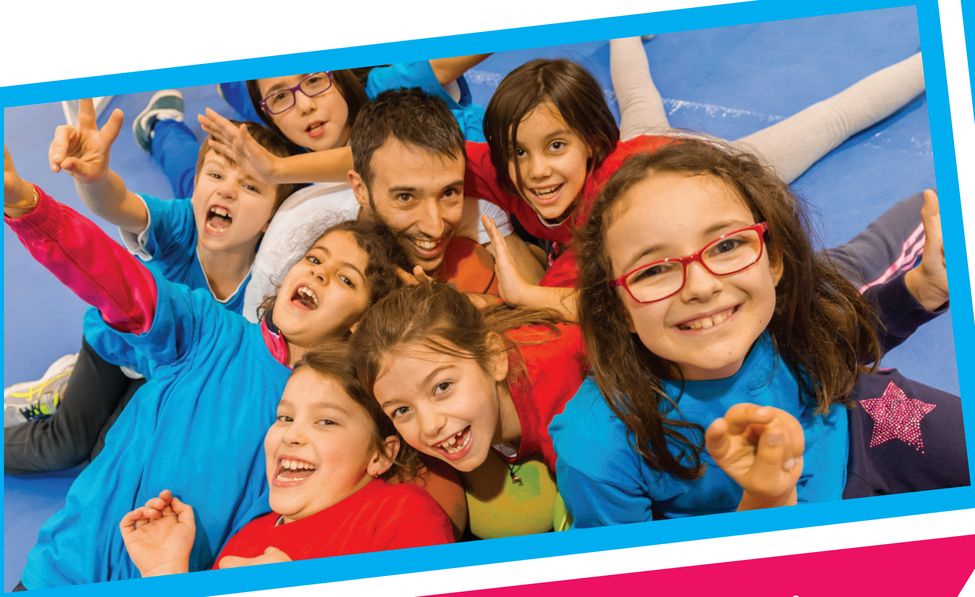

MindMovers & ImaginAction

Playing with variability
to promote motor, cognitive
and citizenship development

Edited by
**Caterina Pesce, Rosalba Marchetti,
Anna Motta, Mario Bellucci**

CALZETTI MARIUCCI
editori

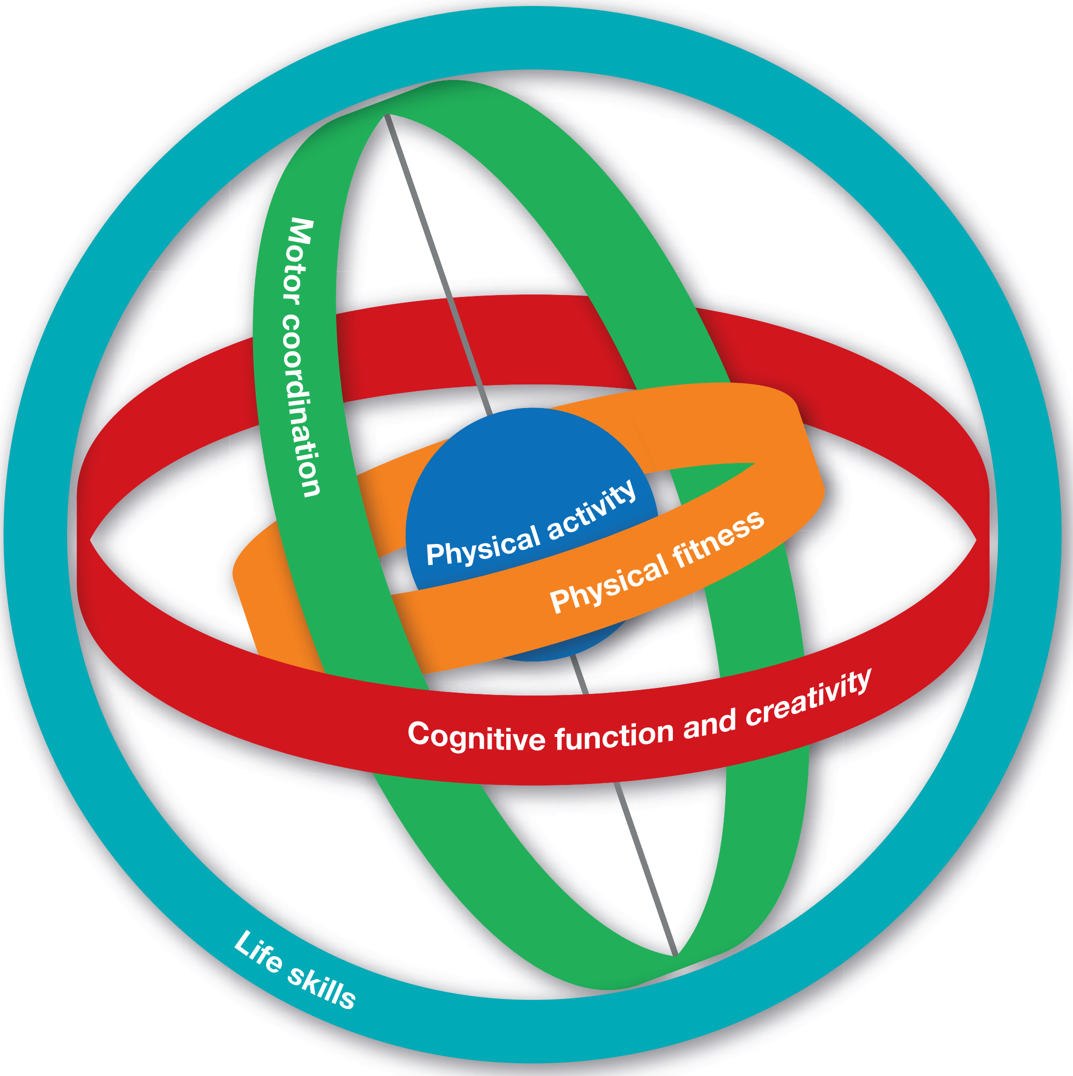

Motor coordination
Physical activity
Physical fitness
Cognitive function and creativity
Life skills

54 THE PHOTOCOPY

www.joyofmovinghandbook.com/en/games/54-photocopy

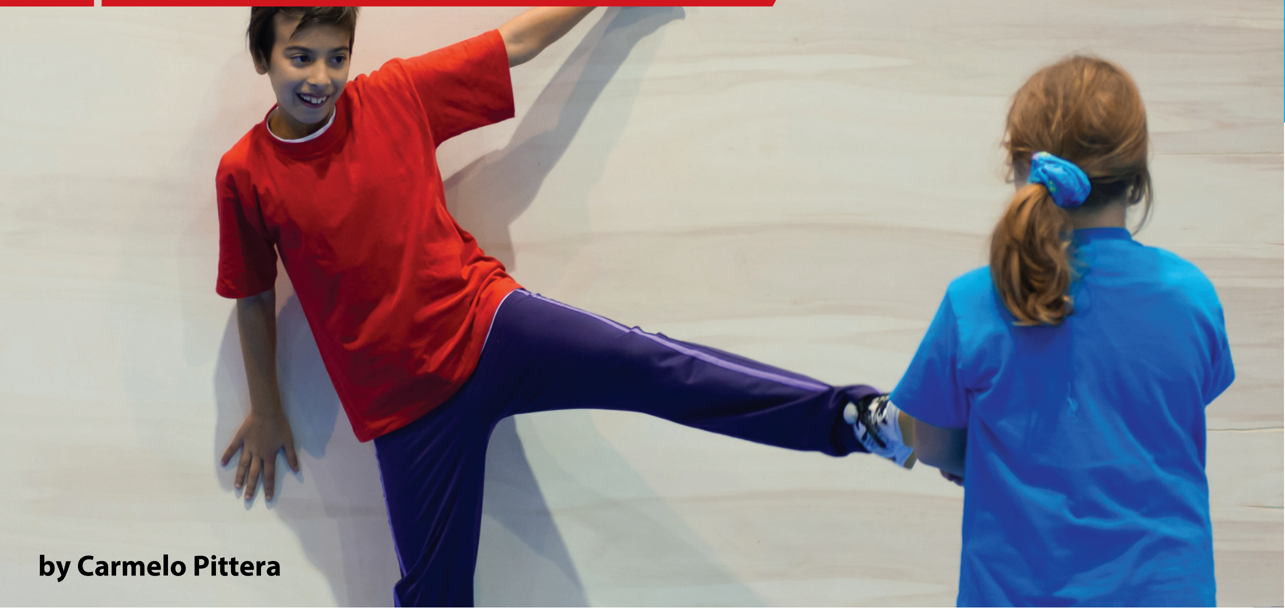

by Carmelo Pittera

Age group

From **6** years upwards

What you need

A wall, balloons or other small pieces of equipment.

How to play

The children must copy the position assumed by the educator or by the playmate in various postures.

Before starting the game

Lie down in a certain position, feeling how the different parts of the body touch the ground, get up and reproduce the position while standing upright. Draw the body on a sheet of paper in a certain position and reproduce it in different postures. In pairs: one child lies down in a certain position on a large piece of paper, the other draws the outline of the body. The first child gets up and the second child observes the figure and tries to reproduce it first while on the ground and then standing up.

Possible variations

- Change movement.
- Increase the coordinative demands.
- Change position.
- Use asymmetrical or asynchronous movements.
- Give free rein to the children's creativity.

Aim of the game

This memory game refers to the knowledge of the body (body schema), including its distal parts. After having observed a movement demonstrated by the educator or a playmate on a spatial plane, the child reproduces the movement on other planes and starting from different positions of the body, elaborating her own mental representation of the movements and positions.

How to play - Instructions for use

The educator explains and demonstrates the exercise against the wall: standing with her forehead against the wall and arms stretched down by her sides, palms resting against the wall. The educator slowly slides her arms up the wall maintaining contact with the wall and rising on tiptoes. She then lowers her arms and returns her heels to the ground.

After demonstrating the exercise, the educator asks the children to repeat it two or three times. She then invites the children to move about for a time ranging from 15 to 60 seconds, freely, or based on a guided activity, depending on the equipment available: gaits, simple games, etc.

After the individual interpretation, the children get into pairs: one performs the exercise against the wall, while the other makes sure that the movement corresponds to the educator's demonstration.

The children once again move about freely, for a time ranging from 15 to 60 seconds, then repeat the exercise by changing the spatial plane: performing the operation on the ground, first in a supine position (lying on their backs), making sure that the backs of their hands are resting on the ground, and that their ankle position is the same as the educator's when she was in an upright position, on tiptoes and with her heels on the ground.

The same exercise is performed in the prone position (lying on their stomachs). The educator checks the adaptation of the motor scheme to the other planes.

Finally, the children are once again organised in pairs: one child makes a figure against the wall, her playmate copies it, while the first child corrects any mistakes. The same exercise can also be done with their backs to the wall.

Once they have mastered the movement, one child proposes to another, as the educator previously did, to perform the movements on the ground while lying prone, supine or on her side, if the figure against the wall is represented with the right or left side leaning on the actual wall.

The children then switch roles.

How to develop the game
From repetition to change

A Repeat the exercise with a new movement, following the same steps described above. The educator is standing up, with her forehead against the wall and arms out with palms resting against the wall. She flexes her right leg, trying to raise it as high as possible, with the tip of her foot pointing downwards. She then repeats it with the opposite leg.

B Repeat the exercise, increasing the coordinative demands. The educator is standing with her forehead against the wall and arms by her sides, palms resting on the wall. She lifts her arms up to the sides and raises them, at the same time, flexing her right leg, trying to keep her knee and ankle (internal malleolus) in contact with the wall. Then she returns to the starting position. The exercise is repeated with the other leg. The children repeat the exercise against the wall and then follow the stages of the game described in the 'Instructions for Use' section.

C Repeat the exercise with one side against the wall: the educator places her left side against the wall, stretching her arms forward and the back of her left hand resting on the wall. She performs a complete circle, upwards and backwards with the arm that is not resting against the wall, rising on her toes and then returning to the starting position. She repeats the exercise leaning her right side against the wall. The children repeat the exercise against the wall and carry out the various stages of the game described in the 'Instructions for Use' section.

D Repeat exercise C with a different movement. The educator stands with her right side against the wall. Her right arm is stretched up, her left arm is stretched out in front of her, with both palms resting on the wall. The child must reproduce the movement by bending his knees as low as possible and repeating this movement two or three times. The exercise is then done a second time after having changed the side leaning against the wall. The children repeat the

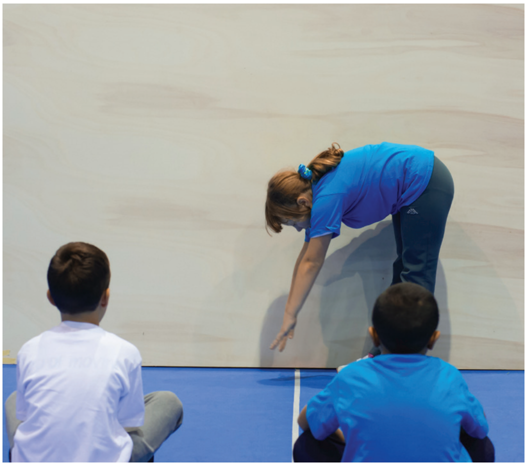

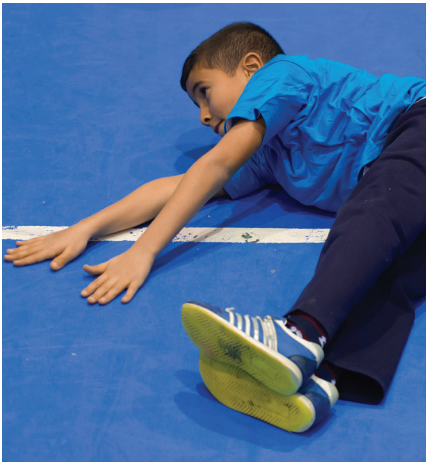

exercise against the wall and then follow the stages of the game described in the 'Instructions for Use' section.

E Repeat the exercise with another movement, this time invented by the children.

How to make the game work - Teaching strategies

The movements suggested here are mere examples: the educator can and should choose other movements and organize drills based on the children's motor abilities and **motor engrams**[1], which she can develop, depending on the level of their skills and abilities. The exercise process is very important: when performing the task with the side leaning against the wall or on the ground, it is interesting to see if the children remember which side they started on. This activity should be followed by a moment of reflection to help them memorize what they have just done, describing the different positions experimented; to fix the sequence in their minds, the educator may ask the children to draw it.

Getting inside the game

Physical fitness objectives

Improving joint flexibility and muscle strength (variations A, B and D).

Motor coordination objectives

Improving accurate motor control abilities in reproducing movements - in particular, interlimb coordination (variations B and D), orientation and precise perceptual-motor adaptation abilities - particularly motor transformation to orient oneself on different spatial planes.

Cognitive and creativity objectives

Improving attention and memory. The game activates Gestalt memory. This is a complex memory task, because to perform the delayed imitation of a new movement, the child must continually update the information held in his working memory.

Life skills objectives

Intrapersonal: improving self-regulation.
Interpersonal: improving social skills (working in pairs).

Keywords

1 - **Motor engram:** a biological memory trace, organized in the parts of the nervous system responsible for memory, as a result of motor learning processes.

55 LITTLE CARS

www.joyofmovinghandbook.com/en/games/55-little-cars

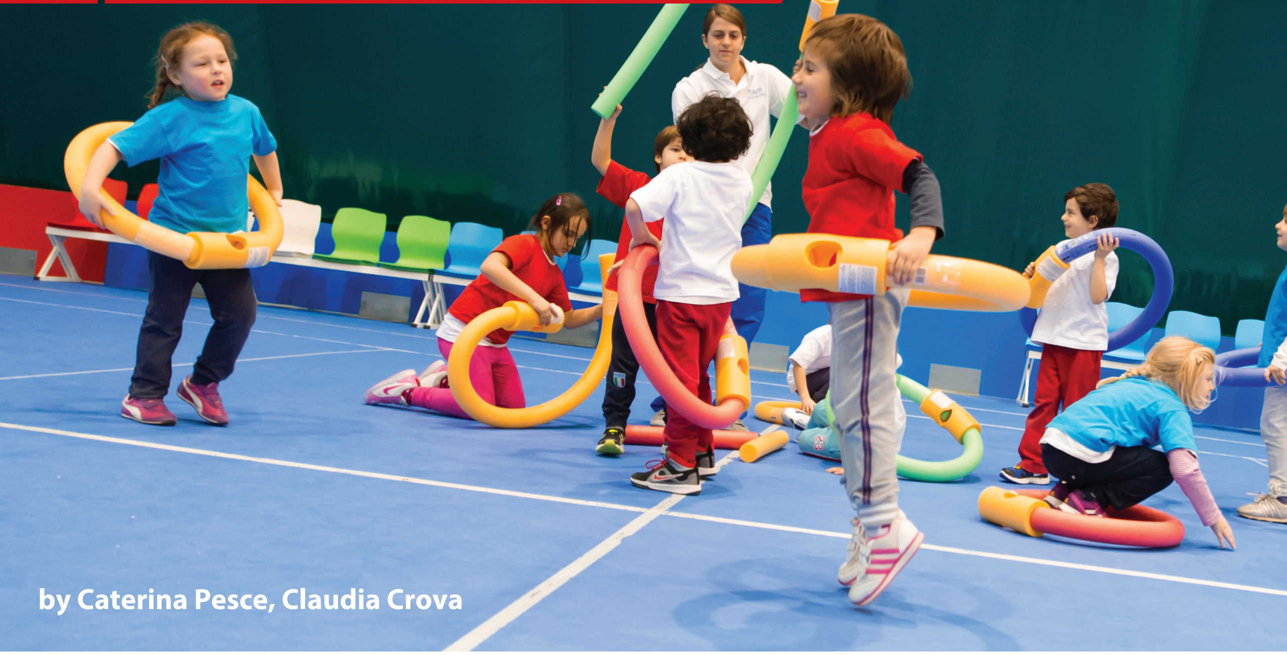

by Caterina Pesce, Claudia Crova

Age group

From **4** years upwards

What you need

Foam mat with holes, long tubes of 3 colors (red, green yellow), short tubes and connectors to make the colored circles of the traffic lights and steering wheels.

How to play

The traffic generated by the car-children is controlled by the traffic officer-educator who operates the traffic light.

Possible variations

- Change color-action association.
- Change the commands corresponding to the position of the traffic officer's arms.
- Create a fairy tale, using color-animal associations.

Before starting the game

The children construct their own cars, using tubes of different lengths and connectors to make a circle. After having explained the basic rule of the game, and before involving all the children, give a demonstration with a minimum number (three) of children.

Aim of the game

The cars game is a motor and cognitive activation game, which involves compliance with the rules that every motorist and pedestrian should know. It fosters correct road and civic education with respect for the Highway Code.
In this activity, the traffic officer-educator controls the traffic of the car-children, coordinating the movements with the use of different colored tubes (yellow, green, red).

How to play – Instructions for use

The educator prepares the game environment, providing a spacious, obstacle-free area for movement. Each child gets into a tube circle, and using their hands pretends to be driving a little car. The educator asks the children to move around using all the space available, using various trajectories, at different speeds and with different gaits (walking, running, galloping, hopping ...) and avoiding collisions.
To indicate the various actions to the car-children, the educator alternates three different colored tubes that represent the colors of traffic lights: when the educator signals with the green tube, the children are free to run or move around in another way; when the color changes, in other words, an interference, the children have to change speed, adjusting to the color on the traffic light: green = all clear; yellow = slow down; red = stop.
The children who cause accidents are penalized: they have to stop and get out of their circle. When the traffic light changes color again, the children get back into their circle and continue playing.

How to develop the game
From repetition to change

A When the traffic officer raises his arms, the car-children slow down and raise the circle as if they were a double-decker bus. When the traffic officer stretches his arms out to the side, the car-children stop and place the circle on the ground. When the traffic officer lowers his arms, the children get out of the circle and, using it as a steering wheel, run as if they were racing cars.

B The traffic officer signals stop by holding the red tube out to the side; he indicates they must slow down by raising the yellow tube up in the air; and lowering the green tube means all clear.

C The traffic officer shows the three tubes of different colors alternately and randomly by moving his arms in all three directions already used (up in the air, out to the side, down low), which the children had learned to react to by slowing

down, stopping or running. Now it is the color of the tube that guides the action, regardless of the position of the traffic officer’s arms.

D The educator can ask the children to pretend to be an animal that is the same color as the traffic light: a frog, associated with the color green, moves by jumping; a lion, associated with yellow moves on all fours, roaring; a butterfly or ladybug, associated with red, takes flight by leaping .

How to make the game work - Teaching strategies

To expand the space used by children and to vary the provenience of the visual stimulus, the educator moves constantly and draws the children’s attention to the color change by associating it with a voice signal.
As soon as the children have learned how the basic game works, the educator can propose more original color-action associations, using the story-telling techniques and imaginative play described in variation D.
Depending on the age of the children, the educator can modulate the amount of time spent on moderate to vigorous activity (**MVPA**[1]) in relation to breaks: with younger children, ‘green light’ runs should be shorter with more frequent breaks. With older children active time and more vigorous activity stages (running) can be increased, reducing the number of breaks and the active time used for more moderate activity (walking).

Keywords

1 - **MVPA:** Moderate-to-Vigorous Physical Activity, corresponds to a heart rate higher than 140 bpm in children

2 - **Stop principle:** applied to tasks where an external stimulus indicates that the person must interrupt an action (e.g. the game “One, two, three... Star!” analyzed in chapter 1).

3 - **Contextual interference:** a phenomenon in which, although the repeated execution of always the same movement brings about the best learning results in the short term, practicing different tasks in random sequence - that is, with interference between different skills - leads to better and more stable long-term learning.

4 - **Self-regulation:** the ability to manage, inhibit or adjust behavior in the interests of the individual and society.

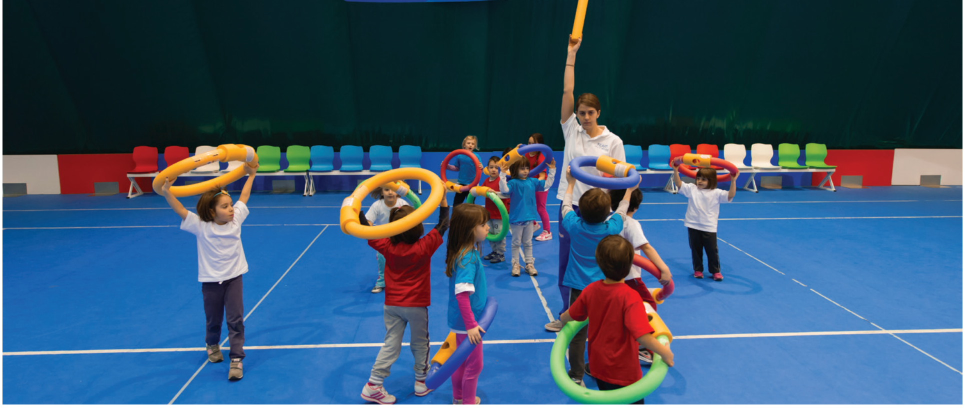

Getting inside the game

Physical fitness objectives

Improving cardiovascular fitness and speed.

Motor coordination objectives

Improving orientation in space, as there are no pre-defined roads and lanes and the car-children are encouraged to find different trajectories. Improving perceptual-motor adaptation abilities, in particular the reaction ability to visual stimuli given by the educator with the tubes, and motor transformation abilities, as the children have to constantly change their routes to adapt to each other's trajectories, which are not defined and therefore unpredictable.

Cognitive and creativity objectives

Improving inhibition, in other words, the ability to brake or interrupt the execution of an action following an unpredictable command, which is never sequential and repetitive (application of the **stop principle**[2]).

As the children gradually learn the game, the educator must pass from a fixed alternation of the traffic lights (green ↦ yellow ↦ red ↦ green… at regular intervals) to a random alternation (e.g. red ↦ green ↦ red ↦ yellow) at varying time intervals, both longer and shorter. In variation C of the game, the **contextual interference**[3] is added to the 'stop' principle, which further trains the inhibitory ability. In fact, the children receive two signals that are inconsistent with each other (position of the arms and tube color), but must react to only one of the two (color of the tube), ignoring the other that serves as a distracter (position of the arms).

In this way they learn to resist the tendency to repeat a previously automated behavior in response to known signals (traffic offer's arms), to switch and act according to other signals (tube color).

Life skills objectives

Intrapersonal: improving **self-regulation**[4].

Interpersonal: improving the ability to communicate in order to avoid collisions.
